# Salphage: Salvage Bacteriophage Therapy for Recalcitrant MRSA Prosthetic Joint Infection

**DOI:** 10.3390/antibiotics11050616

**Published:** 2022-05-04

**Authors:** James B. Doub, Vincent Y. Ng, Myounghee Lee, Andrew Chi, Alina Lee, Silvia Würstle, Benjamin Chan

**Affiliations:** 1 Division of Clinical Care and Research, Institute of Human Virology, University of Maryland School of Medicine, Baltimore, MD 21201, USA; 2 Department of Orthopedic Surgery, University of Maryland School of Medicine, Baltimore, MD 21201, USA; vng@som.umaryland.edu; 3 Department of Pharmacy, University of Maryland Medical Center, Baltimore, MD 21201, USA; mlee1@umm.edu (M.L.); andrew.chi@umm.edu (A.C.); 4 Department of Ecology and Evolutionary Biology, Yale University, New Haven, CT 06520, USA; a.lee@yale.edu (A.L.); silvia.wuerstle@yale.edu (S.W.); b.chan@yale.edu (B.C.); 5 Yale Center for Phage Biology & Therapy, Yale University, New Haven, CT 06520, USA

**Keywords:** bacteriophage therapy, *Staphylococcus aureus*, prosthetic joint infection, cell surface receptor, transaminitis

## Abstract

Prosthetic joint infections are a devastating complication of joint replacement surgery. Consequently, novel therapeutics are needed to thwart the significant morbidity and enormous financial ramifications that are associated with conventional treatments. One such promising adjuvant therapeutic is bacteriophage therapy given its antibiofilm activity and its ability to self-replicate. Herein we discuss the case of a 70-year-old female who had a recalcitrant MRSA prosthetic knee and femoral lateral plate infection who was successfully treated with adjuvant bacteriophage therapy. Moreover, this case discusses the importance of propagating bacteriophage therapeutics on bacteria that are devoid of toxins and the need to ensure bacteriophage activity to all bacterial morphologies. Overall, this case reinforces the potential benefit of using personalized bacteriophage therapy for recalcitrant prosthetic joint infections, but more translational research is needed to thereby devise effective, reproducible clinical trials.

## 1. Introduction

Prosthetic joint infections (PJIs) are the most feared complication of joint replacement surgeries with an estimated 1 to 2% of all knee and hip prosthetics becoming infected during the life of the prosthetic [1]. The gold standard treatment of chronic PJIs is two stage revision surgery whereby the prosthetic is removed and reimplantation of a new arthroplasty is not conducted until after six weeks of antibiotic therapy. This procedure is taxing to patients and has immense financial ramifications [2]. Obstinately, the success rates of this surgical technique have not changed over the past several decades and, consequently, novel therapeutics are drastically needed [3].

One such novel therapeutic for PJI treatments is bacteriophage therapy given these viruses have evolved with bacteria to possess innate anti-biofilm activities, can infect metabolically reduced bacteria and can degrade the biofilm matrices [4]. In addition, bacteriophages can self-replicate on their bacterial hosts, thereby, potentially increasing their numbers to help cure the infection. Therefore, bacteriophages have been proposed to be possible adjuvants with debridement and implant retention surgery to, thereby, cure PJIs without prosthesis removal [5]. However, bacteriophages also hold promise as an adjuvant in revision surgeries when retention of prosthetics is not feasible. Herein we discuss a case of a patient who had a recalcitrant methicillin resistant *Staphylococcus aureus* (MRSA) PJI of her knee and femoral plate that conventional treatments were not able to cure. Rather, only after adjuvant bacteriophage therapy was a sustained clinical and microbiological cure achieved

## 2. Case

**Figure 1:**
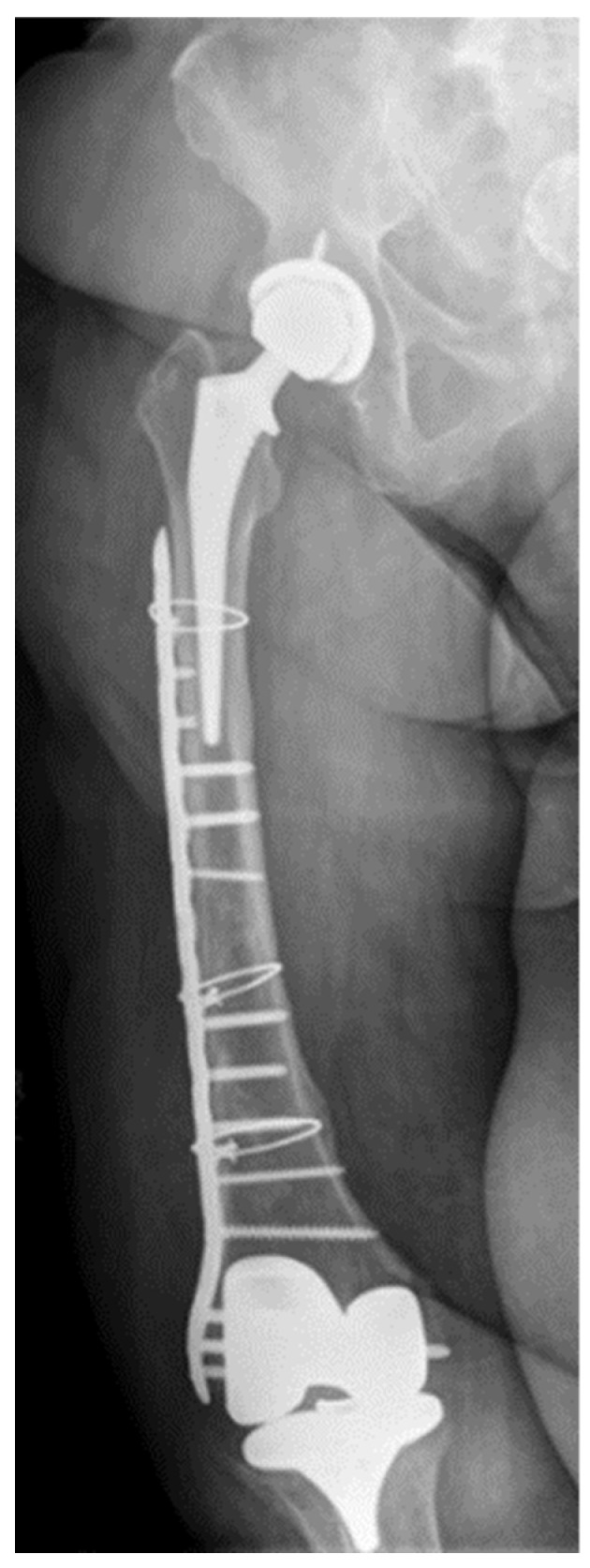
Knee and hip prosthetics with lateral plate with three cerclage wires.

A 70-year-old female with a past medical history of hypertension, diabetes and chronic lymphedema of the right leg presented to the University of Maryland for a second opinion of her recalcitrant MRSA PJI. The patient initially underwent bilateral knee arthroplasties in 2008 for progressive osteoarthritis. Unfortunately, her right knee arthroplasty was complicated by an MRSA PJI requiring revision surgery and intravenous vancomycin and then indefinite oral suppression doxycycline therapy given her chronic lymphedema. She then underwent right hip arthroplasty for progressive osteoarthritis, but this was complicated by a periprosthetic femur fracture requiring the insertion of an extensive lateral femoral plate, cerclage wires and single staged revision of right knee arthroplasty for MRSA PJI followed by 6 weeks of vancomycin therapy and then indefinite oral doxycycline therapy (Figure 1). Three months later, she had worsening pain in her knee and a draining sinus tract. Arthrocentesis culture again grew MRSA. A repeat revision surgery was deemed unlikely to be successful given her chronic lymphedema and extensive chronic MRSA infection. Subsequently, she was recommended for fusion or amputation.

Given the sinus tract and the extent of hardware infection (knee prosthetic, lateral plate and hip prosthetic) in correlation with erosion of the medial condyle, salvage of her prosthesis was not feasible as the prosthetic was loose. Therefore, explantation of all hardware followed by reconstruction with a right revision hip replacement and knee replacement with total femur implant was deemed the best chance to eradicate the infection, salvage her lower extremity and allow for ambulation. However, given the extent and recalcitrant nature of her infection, adjuvant bacteriophage therapy was discussed with this patient. She agreed to this experimental therapy and a repeat arthrocentesis of her knee was obtained. Again, only MRSA was cultured, and the clinical isolate was sent to Dr. Benjamin Chan to create a personalized bacteriophage therapy.

Her clinical isolate was matched to the bacteriophage Mallokai, which had adequate growth inhibition and plaque formation. This bacteriophage was then amplified on her clinical isolate to titers of 1 × 10^10^ PFU/mL. Evaluation of the bacteriophage therapeutic did not reveal any endotoxins and USP-71 sterility testing was negative. Expanded access was granted by the FDA (IND 27250) and approval by the University of Maryland of Baltimore Institutional Review Board (HP-00094883EA) was obtained.

**Figure 2:**
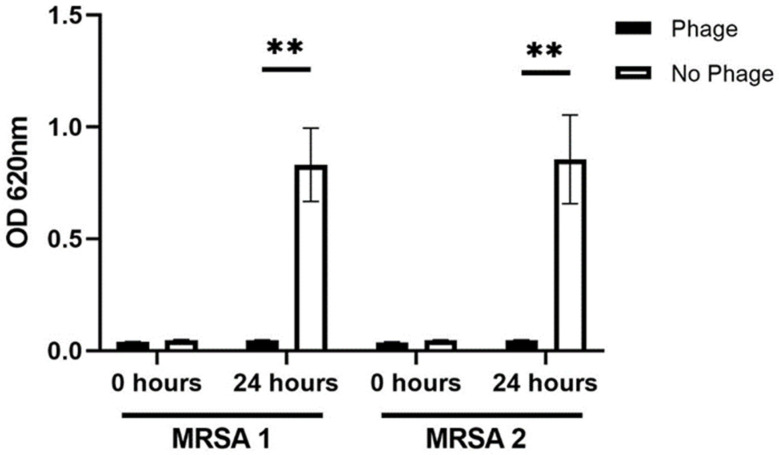
Bacteriophage growth inhibtion assays for the two different MRSA clinical isolates: Significant bacteriophage (Mallokai) induced growth inhibition was observed for both MRSA clinical isolates at 24 h (Wilcoxon test, ** *p* < 0.005). Experiment was conducted with six replicates and was reproduced in triplicate. Error bars are SD.

She then underwent removal of her knee prosthetic, lateral plate and hip prosthetic. At the end of the surgery, an intraoperative dose of bacteriophage (1 × 10^10^ PFU/mL) was diluted in 10 mL of normal saline, with resulting titers administered being 1 × 10^9^ PFU/mL. Intravenous daptomycin, 500 mg every day, was started after the operation. The next day intravenous bacteriophage therapy (1 × 10^10^ PFU/mL) was diluted in 50 mL of normal saline and infused over 30 min, with resulting titers administered being 2 × 10^8^ PFU/mL. She received three doses of intravenous bacteriophage therapy and was planned to receive two more days of intravenous bacteriophage therapy. However, before her fourth dose she developed an increase in aspartate and alanine transferase up to 2.5 times the upper limit of normal and further doses were held. Four days after stopping bacteriophage her transaminitis returned to normal. Operative cultures grew two different distinct MRSA colony morphologies that were spatially separated, with one distinct colony morphology obtained from knee tissues and another colony morphology from the proximal end of the femoral lateral plate adjacent to the distal hip prosthetic. We retrospectively tested both morphologies to ensure bacteriophage activity (Figure 2).

**Figure 3:**
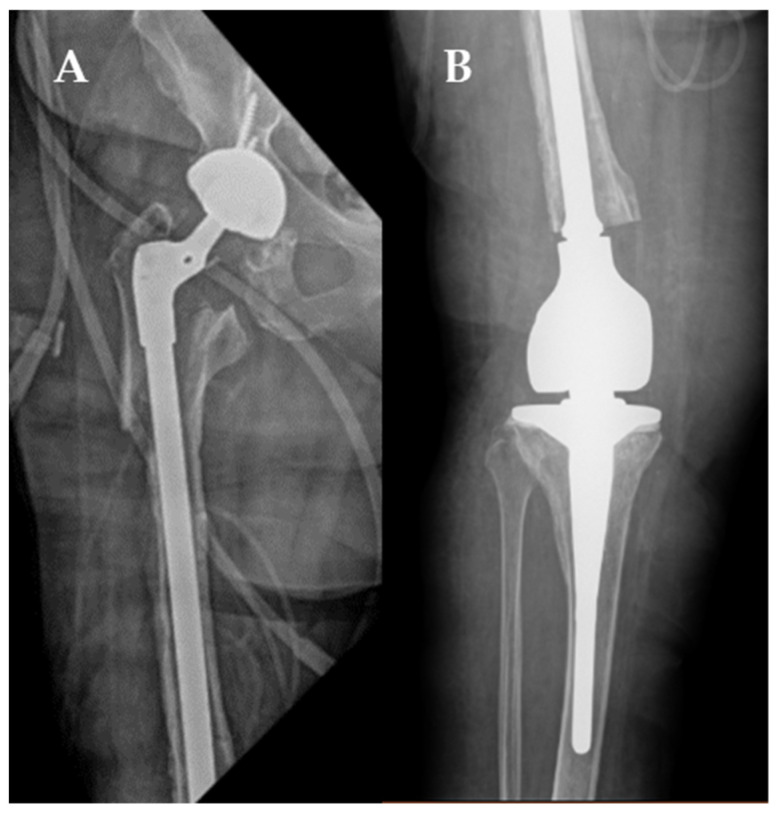
After cure of recalcitrant MRSA PJI and implantation of right total hip arthroplasty (**A**) extending in continuity with a total knee arthroplasty (**B**).

She then completed six weeks of intravenous ceftaroline therapy, 600 mg every 12 h, as daptomycin was cost prohibitive. After six weeks, all antibiotics were stopped, and after two months off antibiotic therapy, a repeat arthrocentesis was obtained that showed no evidence of infection in the hip or knee joints. One month later, she underwent reconstruction of her hip and knee joints with an intramedullary total femur implant (Figure 3). No MRSA could be cultured from operating room cultures and no infection was seen in the operating room, but a rare growth of Streptococcus pyogenes was seen in one culture. While this was thought to be a contaminate, especially given the lack of any signs of infection in the operating room, we elected to treat for four weeks with intravenous ceftriaxone therapy, 2 g every day, to be conservative. In addition, as a result of her chronic lower extremity lymphedema and the extensive reconstruction, we elected to use oral Cephalexin, 500 mg twice daily, to prevent further PJIs after the ceftriaxone therapy. Given the prolonged period in which she had not ambulated, she is currently receiving rehab to increase her mobility but has had no signs of recurrence of her infection 12 months later.

## 3. Discussion

Two stage revision surgery is the gold standard for the treatment of chronic PJIs, but this intervention is associated with significant morbidity and financial ramifications [1,2]. When PJIs fail, two stage revision surgery limited standardized options are available. This is in part because the biofilm laden prosthetic was removed and, consequently, all theoretical niduses of infection were eliminated. Therefore, when immunocompetent patients have recurrence with the same pathogen, this suggests that a deep-seated infection may be present. Bacterial factors that cause these deep-seated infections include: persister cells, small colony variants, plasma protein aggregates and microscopic abscesses in cortical canaliculi [6,7,8]. Furthermore, when additional infected hardware is present beyond the infected prosthetic, this complicates treatment and makes eradication even more difficult as seen here.

In this case the patient had failed standard of care revision surgeries and a draining sinus tract was present over her knee, indicating a chronic MRSA infection. A previous periprosthetic femur fracture required a large lateral plate that was also infected with MRSA and, obstinately, this was directly adjacent to the distal end of her hip prosthetic. Unfortunately, her hardware could not be salvaged given the instability of the knee prosthetic, but it was paramount to cure her infection to thereby implant a megaprosthesis to give her the best chance to ambulate again on her lower extremity (Figure 3). Therefore, the use of intraoperative and intravenous bacteriophage therapy was used as an adjuvant to help sterilize the joint. The benefits of using bacteriophage therapy with surgical interventions have been discussed and have been used by other researchers [5,9,10,11]. Moreover, surgical interventions are a central dogma of PJI treatments to achieve infection source control. No standard of care PJI treatment is recommended without some form of surgery [12,13]. However, the use of surgical intervention does hinder the ability to definitively prove effectiveness of the bacteriophage therapeutic, but in this case the intraoperative cultures off antibiotics for many months supports sterilization of her infection. However, only randomized clinical trials will be able to definitively prove if using bacteriophages as therapy are effective adjuvants in PJI treatments.

While the bacteriophage therapy helped cure her recalcitrant MRSA infection, we did observe a mild transaminitis with our protocol of intraoperative phage and then intravenous phage therapy. As seen in a previous reported case, it was not until after the third intravenous dose that we observed a mild transaminitis, which further supports the need for close monitoring of liver enzymes when using bacteriophage therapy for PJIs [14]. Additionally, since her bacteriophage therapeutic was grown on her MRSA clinical isolate, we retrospectively evaluated her therapeutic for possible enterotoxins in which we observed low levels of Staphylococcal enterotoxin A (3 ng/mL). It is unknown if this low level of enterotoxin caused her to have mild liver inflammation or if the inflammation was cytokine driven for reasons we state elsewhere [14,15]. Nonetheless, this case reinforces that bacteriophage therapies can be contaminated with other elements beyond endotoxins and supports the need to grow bacteriophage therapeutics on bacteria that are devoid of these contaminants to mitigate the potential risks [15].

What was also interesting was that the patient had two distinct different MRSA clinical isolates, which had different colony morphologies. Nonetheless, given the narrow spectrum of bacteriophage activity, the bacteriophage used here was tested to ensure growth inhibition to both morphologies. Figure 2 shows the results in which no differences in growth inhibition were seen for the two morphologies. Even though the bacteriophage had activity to both isolates, this case reinforces the need to ensure bacteriophage activity to all isolates and morphologies given that assuming activity is what has led to failed clinical trials [16]. Furthermore, most Staphylococcal bacteriophages bind to teichoic acid, but this receptor can have different glycosylation patterns in different environments, which can have ramifications for bacteriophage activity [17,18,19]. Therefore, at this nascent stage when using bacteriophages for PJI, there is ample evidence supporting bacteriophage therapy use as an adjuvant to surgical interventions instead of in lieu of surgery. This is to thereby allow for effective and reproducible treatments as well as to isolate all bacteria and all potential phenotypes of the causative bacteria to ensure activity of the bacteriophage therapeutic.

## 4. Materials and Methods

### 4.1. Bacteriophage Screening, Amplification and Purification

The screening, amplification and purification followed the same procedure as previously described [20]. To note, the concentrated bacteriophage was buffered in plasmalyte. The final therapeutic was quality control tested for titers, sterility (USP <71>) and endotoxin levels. The results from quality control testing can be found in Table 1. Retrospectively, we also tested for levels of Staphylococcal enterotoxin A-C with use of commercially available ELISA testing (Thermo Fisher Scientific, Waltham, MA, USA), which can be seen in Table 1.

### 4.2. Testing for Bacteriophage Activity to the Two MRSA Morphologies

Overnight cultures of the two MRSA morphologies were grown in tryptic soy broth (TSB) to optical density 0.30–0.60, representing exponential growth (OD 620 nm). Bacterial cultures were then diluted to OD of 0.024 with TSB and placed into wells of microtiter plates. Negative control included TSB without bacteria or bacteriophages (not shown in Figure 2), but no changes in OD were seen. Positive control included bacteria in TSB without bacteriophages. Wells of bacteria in TSB were infected with 0.05 mL of bacteriophage with the same titers as used for the patient (1 × 10^10^ PFU/mL). OD was read at time zero and again after microwell plates were incubated at 37 degrees Celsius for 24 h. Results were reproduced in triplicate.

## 5. Conclusions

In conclusion, this case adds to the growing data supporting the potential use of bacteriophage therapy as an adjuvant to surgical interventions in PJI treatment. Bacteriophage therapy may be a promising agent in treating PJI to either circumvent the need for revision surgery or to enhance the efficacy of revision surgery, especially in complex cases that have high risk of recurrence. However, more translational research is needed to clarify many aspects of this therapeutic to devise effective, reproducible protocols before efficacy clinical trials are conducted.

## Figures and Tables

**Table 1:** Titer, endotoxin, sterility and exotoxin levels of the bacteriophage used in this case.

Phage ID	Titer (PFU/mL)	Endotoxin (EU/Dose)	USP <71> Sterility	Staphylococcal Enterotoxin A (ng/mL)
Mallokai	1 × 10^10^	<1	No Growth	3

## Data Availability

Not applicable.
